# Genomic insights into the 2020 mass die-off event among African elephants

**DOI:** 10.1038/s41467-025-63446-7

**Published:** 2025-09-26

**Authors:** Astrid Rasmussen, Louise Roer, Øystein Angen, Marijke M. Henton, Magne Bisgaard, Henrik Christensen, Jesper Larsen

**Affiliations:** 1 Department of Bacteria, Parasites & Fungi, Statens Serum Institut, Copenhagen, Denmark; 2 Vetdiagnostix, Blue Hills, Midrand, South Africa; 3 Bisgaard Consulting, Viby Sjælland, Denmark; 4 Department of Veterinary and Animal Sciences, University of Copenhagen, Frederiksberg, Denmark

**Keywords:** Bacterial pathogenesis, Bacterial evolution

**arising from** C.M. Foggin et al. *Nature Communications* 10.1038/s41467-023-41987-z (2023**)**

The article ‘*Pasteurella* sp. associated with fatal septicaemia in six African elephants’ by Foggin et al.^1^ shows that the mass die-off event involving 35 elephants in north-western Zimbabwe during the hot dry season (August–November) of 2020 was likely due to haemorrhagic septicaemia caused by sucrose-negative *Pasteurella multocida*-like bacteria belonging to Bisgaard taxon 45. VF20HR from elephant VF20/112 represents the only sequenced Bisgaard taxon 45 genome, which prevented Foggin et al.^1^ from investigating the phylogenetic relationships and genetic differences between the outbreak strain and other members of Bisgaard taxon 45. Here, we reconstruct annotated genomes of ten additional Bisgaard taxon 45 isolates in order to identify virulence factors present in the outbreak strain but not in conspecific isolates from other hosts. The results show that the outbreak strain contains a unique combination of toxins and surface antigens that could explain the observed invasiveness and pathogenesis.

Members of Bisgaard taxon 45 asymptomatically colonise the mucosal surface of the oropharynx of lions, leopards, and tigers and are the causative agent of serious infections in people bitten by these animals, but they have not been previously reported as a cause of haemorrhagic septicaemia in African elephants^2^. Foggin et al.^1^ were able to assemble the first draft of a Bisgaard taxon 45 genome (VF20HR) through Illumina shotgun sequencing of liver and spleen samples collected from elephant VF20/112. BLAST+ searches showed that the VF20HR genome contains 22 of 25 tested *P*. *multocida* virulence factors, including an open reading frame (ORF) encoding a 964-residue protein called PmHAS. Unfortunately, PmHAS was misannotated as a hyaluronidase (also called hyaluronate lyase) in ref. 15 in Foggin et al.^1^, which led the authors to assume that the outbreak strain is capable of producing this enzyme. Hyaluronidase is produced by *P*. *multocida* capsular serogroup B isolates, causing haemorrhagic septicaemia in cattle and buffaloes in Asia^3^, and would indeed have been a striking finding in the outbreak strain. However, the PmHAS ORF (also called *hyaD*) encodes a hyaluronan synthase involved in capsule formation in *P*. *multocida* expressing capsular serogroup A, the causative agent of fowl cholera^4^.

We used digital DNA-DNA hybridisation (dDDH) to infer the taxonomic relationships between the VF20HR genome and type strain genomes in the TYGS database^5,6^. The results showed that VF20HR is most closely related to the type strains of *P*. *multocida* subsp. *multocida*, *gallicida*, and *septica*, thus confirming previous results based on sequence analysis of 16S rRNA^1,2^. The dDDH values are listed in Supplementary Data 1.

We used Illumina technology to sequence the two Bisgaard taxon 45 isolates that were identified in the brain and liver samples from elephant VF20/112 (i.e. the same elephant from which the VF20HR genome was reconstructed) by Foggin et al.^1^, six Bisgaard taxon 45 isolates from other sources, and four *Pasteurella*-like isolates collected from two cheetahs, a lion, and a Nguni calf in South Africa. We also sequenced the closely related type strains of *P*. *multocida* subsp. *multocida*, *gallicida*, and *septica*, which express capsular serogroup A and represent the two major clades—*P*. *multocida* subsp. *multocida*/*gallicida* and *P*. *multocida* subsp. *septica*—within a recently published phylogeny of a *P*. *multocida* reference collection encompassing capsular serogroups A, B, D, E, and F^7^. Finally, we sequenced eight sucrose-negative capsular serogroup A isolates belonging to *P*. *multocida* subsp. *multocida* (*n* = 4) and *P*. *multocida* subsp. *septica* (*n* = 4) and downloaded the genomes of five existing genomes of *P*. *multocida* expressing capsular serogroup E, the causative agent of haemorrhagic septicaemia in cattle and buffaloes in Africa^8^; these isolates were selected because the former share phenotypic traits (unable to utilise sucrose) with Bisgaard taxon 45 and the latter share geographical range with the outbreak strain and cause similar pathological lesions. Detailed information on the Bisgaard taxon 45 and *P*. *multocida* genomes used in this study is listed in Supplementary Data 2. Phylogenetic analysis showed that the three isolates from elephant VF20/112, the lion isolate, the calf isolate, and the six Bisgaard taxon 45 isolates clustered together, whereas the two cheetah isolates, the sucrose-negative *P*. *multocida* subsp. *multocida* and *septica* capsular serogroup A isolates, and the *P*. *multocida* subsp. *multocida* capsular serogroup E isolates formed separate clusters within the *P*. *multocida* subsp. *multocida*/*gallicida* clade (Fig. 1 and Supplementary Data 3). The *P*. *multocida* subsp. *multocida* capsular serogroup E isolates also formed a separate cluster within the *P*. *multocida* subsp. *multocida*/*gallicida* clade in the recently published phylogeny of the *P*. *multocida* reference collection^7^. The sucrose-negative *P*. *multocida* subsp. *septica* capsular serogroup A isolates probably represent sorbitol-negative variants of *P*. *multocida* subsp. *multocida*^2^, which explains the closer relationship to the type strains of *P*. *multocida* subsp. *multocida* and *gallicida* than to the type strain of *P*. *multocida* subsp. *septica*. The three elephant VF20/112 isolates formed a late-branching subcluster within Bisgaard taxon 45 and pairwise single-nucleotide polymorphism (SNP) comparison showed that the isolates were identical to each other (Fig. 1 and Supplementary Data 3).

**Fig. 1:**
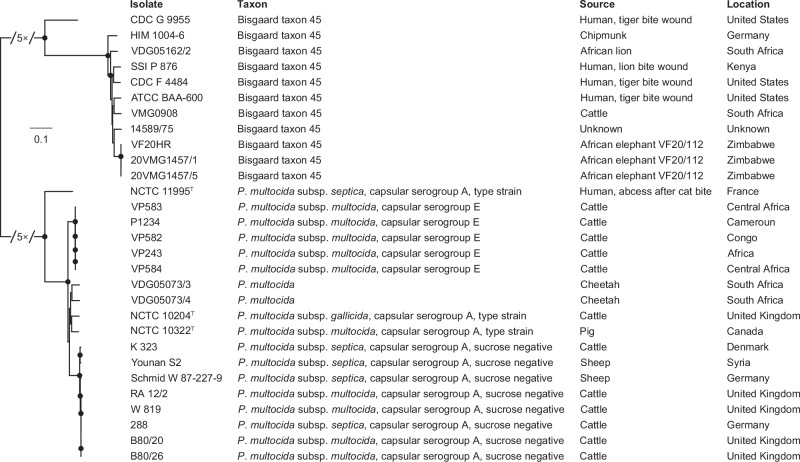
Phylogenetic context of elephant VF20/112 isolates. The three elephant VF20/112 isolates, the lion isolate, the calf isolate, and the six Bisgaard taxon 45 isolates clustered together, whereas the two cheetah isolates formed a separate cluster within the *P*. *multocida* subsp. *multocida*/*gallicida* clade. The three elephant VF20/112 isolates formed a late-branching subcluster within Bisgaard taxon 45. The maximum-likelihood phylogeny was built from a core-genome single-nucleotide polymorphism (SNP) alignment (150,135 SNPs) after putative recombination sites were removed. The length of the broken branches was reduced by fivefold. Branch support values above 90% are indicated by filled circles at the nodes. The scale bar denotes substitutions per variable site.

We performed Nanopore sequencing to reconstruct complete, closed genomes of three Bisgaard taxon 45 isolates, including 20VMG1457/1 from elephant VF20/112, and used Illumina reads to assemble the remaining eight Bisgaard taxon 45 genomes. Analysis of the annotated Bisgaard taxon 45 genomes (Supplementary Data 4–14) showed that the three elephant VF20/112 isolates harboured an intact serogroup A capsule biosynthesis locus, including *hyaD*, with ≈85% nucleotide identity to the locus in *P*. *multocida* X73 (GenBank accession no. AF067175), as well as a nearly intact genotype L6 lipopolysaccharide (LPS) outer core biosynthesis locus (LPS serovars 10, 11, 12, and 15), with ≈84% nucleotide identity to the locus in *P*. *multocida* P1573 (GenBank accession no. KJ689443), except that the pseudogene nat_ps was missing in the three elephant VF20/112 isolates. Interestingly, the hyaluronan polysaccharide capsule and genotype L6 LPS are both considered to be important virulence factors in *P*. *multocida* by reducing phagocytosis and mimicking host glycans, respectively, and it therefore seems reasonable to assume that they have a similar function in the three elephant VF20/112 isolates^9,10^.

The complete, closed 20VMG1457/1 genome from elephant VF20/112 consisted of a circular 2,266,452-bp chromosome. Comparison of the eleven Bisgaard taxon 45 genomes showed that 42 of the 2018 genes present in the 20VMG1457/1 genome existed in only the three elephant VF20/112 isolates (Supplementary Data 15). Five of the 42 genes were carried on a horizontally acquired genetic island, of which two *rtxA*-like genes (DOKOJH_04955 and DOKOJH_04980) encode repeats in toxin (RTX) proteins that are more homologous to the FrpC protein produced by clinical isolates of *Neisseria meningitidis* during invasive disease^11^ than to other known members of the RTX protein family (Fig. 2a). It has previously been shown that physiological concentrations of calcium ions induce cleavage of the peptide bond between residues Asp^414^ and Pro^415^ of FrpC and that the newly generated amino-terminal fragment of FrpC can be covalently cross-linked to another protein molecule^12^. DOKOJH_04955 and DOKOJH_04980 contain most of the segments necessary and sufficient for FrpC processing (residues 400–657 in FrpC corresponding to residues 535–783 in DOKOJH_04955 and residues 166–402 in DOKOJH_04980). The Asp-Pro bond in FrpC was conserved in DOKOJH_04955 (residues 540–541), whereas Asp was replaced by Cys in DOKOJH_04980 (residues 165–166). The *frpC* locus in *N*. *meningitidis* contains the *frpD* gene, which encodes an outer membrane lipoprotein FrpD that binds to the amino-proximal portion of FrpC, whereas it lacks the genes encoding an acyltransferase (*rtxC*) and the type I secretion system (*rtxB* and *rtxD*) that are responsible for posttranscriptional activation and secretion of many RTX proteins, respectively^13^. In comparison, 20VMG1457/1 lacks *rtxC*- and *frpD*-like genes but contains *rtxB*- and *rtxD*-like genes (DOKOJH_04975 and DOKOJH_04970, respectively) (Fig. 2b). It is beyond the scope of this article to provide a detailed review of the remaining genes that existed in only the three elephant VF20/112 isolates, but it is worth pointing out that several of the 42 genes were predicted to be involved in biosynthesis and transport of surface antigens (e.g. glucosyltransferases, outer membrane proteins, fimbriae, pili, and secretion systems), which might have a role in the virulence of the outbreak strain.

**Fig. 2:**
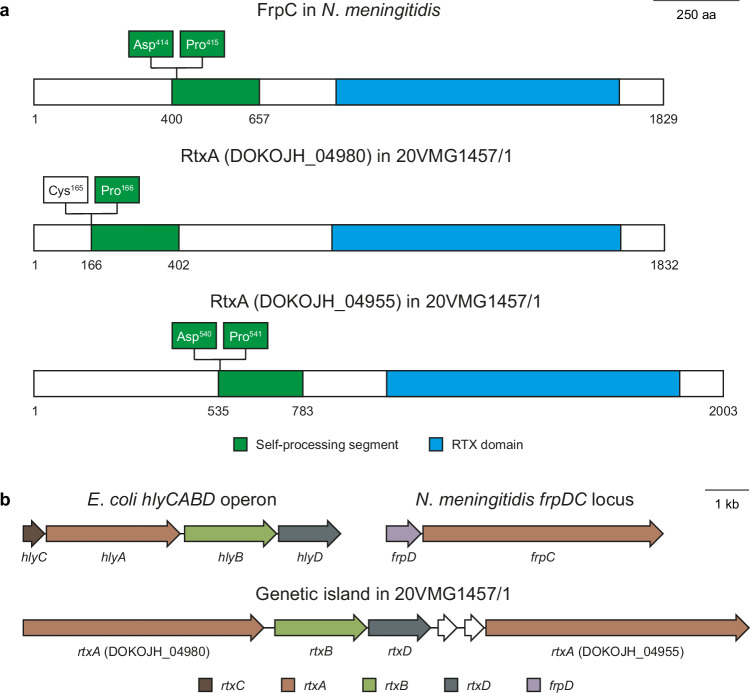
RTX toxins in elephant VF20/112 isolates. **a** Comparison of the *Neisseria meningitidis* FrpC protein (UniProtKB/Swiss-Prot accession no. FRPC_NEIMC) and the two RtxA toxins encoded by DOKOJH_04955 and DOKOJH_04980 in the elephant VF20/112 isolate 20VMG1457/1. **b** Comparison of the archetypal *Escherichia coli* haemolysin (*hlyCABD*) operon (GenBank accession no. M10133), the *frpDC* locus in *N*. *meningitidis* (GenBank accession no. L06299), and the RTX toxin-encoding genetic island (DOKOJH_04980 through DOKOJH_04955) in the elephant VF20/112 isolate 20VMG1457/1. Conserved domains and homologous genes are shown in the same colour.

In conclusion, we show that the three elephant VF20/112 isolates contain a unique combination of toxins and surface antigens, and could therefore possibly represent a hypervirulent clone with potential for causing high rates of invasive disease. Most notably, two of the genes encode homologues of the FrpC protein produced by *N*. *meningitidis*, which is an important cause of life-threatening septicaemia and meningitis in humans^14^. FrpC is only distantly related to RTX toxins found in other Gram-negative bacteria, including members of the family Pasteurellaceae, and its role in the pathogenesis of Bisgaard taxon 45 and *N*. *meningitidis* infection is currently unclear. Interestingly, Bisgaard taxon 45 was isolated in heavy growth from the two elephant brains examined by Foggin et al.^1^, and it is therefore tempting to speculate that FrpC might enable these bacteria to cross the blood-brain barrier. It should be noted, however, that the virulence factors that determine whether invasive disease will develop or not are poorly understood. It has previously been shown that phase-variable type III restriction-modification systems might play a direct role in the pathogenesis of other members of Pasteurellaceae, including *Haemophilus influenzae* and *Mannheimia haemolytica*^15^, raising the possibility that the ability of Bisgaard taxon 45 to invade the bloodstream and cross the blood-brain barrier is facilitated by epigenetic mechanisms such as phase variation through stochastic, reversible switching of gene expression as observed for *N*. *meningitidis*^14^. Future studies should seek to identify the epidemiology of the outbreak strain through screening of the local wildlife populations, including healthy elephants as well as known reservoir hosts such as lions and leopards. Whole-genome sequencing of the collected isolates could then be used to identify the precise repertoire of virulence genes and mutational events involved in mass die-off events caused by Bisgaard taxon 45 and other *Pasteurella* taxa, including the death of more than 200,000 saiga antelopes in central Kazakhstan during the calving aggregation in May 2015^16,17^. These studies will not only increase our understanding of the diversity, evolution, epidemiology, and pathogenesis of different *Pasteurella* organisms but can also be used to design and compare the potential coverage of new vaccine candidates.

## Methods

A detailed description of the methods used in this article is provided in the Supplementary Methods.

### Reporting summary

Further information on research design is available in the Nature Portfolio Reporting Summary linked to this article.

## Supplementary information

Supplementary Information

Description of Additional Supplementary Files

Dataset 1

Dataset 2

Dataset 3

Dataset 4

Dataset 5

Dataset 6

Dataset 7

Dataset 8

Dataset 9

Dataset 10

Dataset 11

Dataset 12

Dataset 13

Dataset 14

Dataset 15

Reporting Summary

## Data Availability

dDDH values between the VF20HR genome and type strain genomes in the TYGS database are listed in Supplementary Data 1. Detailed information on the 29 Bisgaard taxon 45 and *P*. *multocida* genomes used in this study is listed in Supplementary Data 2. Sequence data generated in this study have been deposited in the European Nucleotide Archive/NCBI Sequence Read Archive under BioProject PRJNA1204292, and the accession numbers are provided in Supplementary Data 2. Sequence data from other sources comprised the VF20HR genome (GenBank accession no. JAQAHH000000000.1) and the genomes of the five *P*. *multocida* isolates expressing capsular serogroup E (GenBank accession nos NZ_JAMJVB000000000.1, NZ_JAMJVC000000000.1, NZ_JAMJVD000000000.1, NZ_JAMJVE000000000.1, and NZ_JAMJVF000000000.1). The phylogeny shown in Fig. 1 is provided as a Newick file in Supplementary Data 3. The annotated Bisgaard taxon 45 genomes are provided in GenBank Flat File format in Supplementary Data 4–14. The 42 genes present in the three elephant VF20/112 isolates but absent in the other Bisgaard taxon 45 isolates are listed in Supplementary Data 15.
