# Author Correction: Genomic insights into the 2020 mass die-off event among African elephants

**DOI:** 10.1038/s41467-025-67490-1

**Published:** 2026-02-18

**Authors:** Astrid Rasmussen, Louise Roer, Øystein Angen, Marijke M. Henton, Magne Bisgaard, Henrik Christensen, Jesper Larsen

**Affiliations:** 1 Department of Bacteria, Parasites & Fungi, Statens Serum Institut, Copenhagen, Denmark; 2 Vetdiagnostix, Blue Hills, Midrand, South Africa; 3 Bisgaard Consulting, Viby Sjælland, Denmark; 4 Department of Veterinary and Animal Sciences, University of Copenhagen, Frederiksberg, Denmark

**Keywords:** Bacterial pathogenesis, Bacterial evolution

Correction to: *Nature Communications* 10.1038/s41467-025-63446-7, published online 26 September 2025

In the version of the article initially published, in Fig. 1, in the sixth row of the “Source” column, “ATCC-BAA-600” should have read “Human, tiger bite wound”. In the twenty-fifth row of the “Location” column, “RA-12-2” should have read “United Kingdom”. These corrections have now been made to the HTML and PDF versions of the article.

