# Supplementary material for: Genomic insights into the 2020 mass die-off event among African elephants
Source: Nat Commun. 2025 Sep 26;16:8525. doi: 10.1038/s41467-025-63446-7 (PMC12475079; doi:10.1038/s41467-025-63446-7)
Supplement: Supplementary file 1 — Supplementary Information [file 41467_2025_63446_MOESM1_ESM.pdf]

Genomic insights into the 2020 mass die-off event among African elephants

Supplementary Information

Supplementary information included in this .pdf:

- Supplementary Methods
- Supplementary References

Supplementary information uploaded in addition to this .pdf:

- Supplementary Data Files (1-15)

Supplementary Methods

We used the TYGS server to calculate digital DNA-DNA hybridization values between query and type strain genomes in the TYGS database[1,2]. We sequenced 23 isolates on a NextSeq platform (Illumina) with 2 × 151 bp. Three Bisgaard taxon 45 isolates were also sequenced on a GridION benchtop (Oxford Nanopore Technologies). Base calling was performed using Dorado v.7.2.1 (Oxford Nanopore Technologies). Hybracter v.0.7.3[3] was used for long-read assembly and short-read error correction. Short-read assembly of the 20 remaining genomes was performed with SPAdes v.3.15[4]. The VF20HR genome (GenBank accession no. JAQAHH000000000.1) and the genomes of the five *P. multocida* isolates expressing capsular serogroup E (GenBank accession no. NZ_JAMJVB000000000.1, NZ_JAMJVC000000000.1, NZ_JAMJVD000000000.1, NZ_JAMJVE000000000.1, and NZ_JAMJVF000000000.1) were sequenced in previous studies[5,6]. Mapping of Illumina paired-end reads and single-nucleotide polymorphism (SNP) calling were carried out using NASP v.1.2.0[7] as follows: (1) Illumina paired-end reads were mapped against the closed genomes of *P. multocida* subsp. *multocida* isolate NCTC 10322^T (GenBank accession no. NZ_LT906458.1) with the Burrows-Wheeler Alignment tool[8]; (2) SNP calling was achieved using the GATK Unified Genotyper[9,10], setting depth of coverage and unambiguously base calls to ≥10× and ≥90%, respectively, and ignoring insertions and deletions; and (3) SNPs contained in repeats were excluded using NUCmer[11,12]. Gubbins v.3.3.1[13] was used to remove recombination tracts. Phylogenetic reconstruction was carried out using the maximum-likelihood program PhyML v.3.0 with a GTR + R model of nucleotide substitution and 100 bootstrap replicates[14,15]. Bisgaard taxon 45 genomes were annotated with Bakta v.1.8.2[16], and pan-genomic analyses of genome annotations were performed with Roary v.3.13.0[17]. The 42 genes present in the three elephant VF20/112 isolates but absent in the other Bisgaard taxon 45 isolates were used as queries in RPS-BLAST searches against the Conserved Domain Database v.3.21. The closest relatives of the predicted RtxA proteins were identified by BLASTP analysis against UniProtKB/Swiss-Prot[18].

Supplementary References

1. Meier-Kolthoff J. P. & Göker M. TYGS is an automated high-throughput platform for state-of-the-art genome-based taxonomy. *Nat. Commun.* **10**, 2182 (2019).
2. Meier-Kolthoff J. P., Carbasse J. S., Peinado-Olarte R. L. & Göker M. TYGS and LPSN: a database tandem for fast and reliable genome-based classification and nomenclature of prokaryotes. *Nucleic Acids Res.* **50(D1)**, D801-D807 (2022).
3. Bouras, G. et al. Hybracter: enabling scalable, automated, complete and accurate bacterial genome assemblies. *Microb. Genom.* **10**, 001244 (2024).
4. Bankevich, A. et al. SPAdes: a new genome assembly algorithm and its application to single-cell sequencing. *J. Comput. Biol.* **19**, 455-477 (2012).
5. Foggin, C. M. et al. *Pasteurella* sp. associated with fatal septicaemia in six African elephants *Nat. Commun.* **14**, 6398 (2023).
6. Christensen, H. et al. Prediction of *Pasteurella multocida* serotypes based on whole genomic sequences. *Vet. Microbiol.* **271**, 109492 (2022).
7. Sahl, J. W. et al. NASP: an accurate, rapid method for the identification of SNPs in WGS datasets that supports flexible input and output formats. *Microb. Genom.* **2**, e000074 (2016).
8. Li, H. & Durbin, R. Fast and accurate short read alignment with Burrow-Wheeler transform. *Bioinformatics* **25**, 1754-1760 (2009).
9. McKenna, A. et al. The Genome Analysis Toolkit: a MapReduce framework for analyzing next-generation DNA sequencing data. *Genome Res.* **20**, 1297-1303 (2010).
10. DePristo, M. A. et al. A framework for variation discovery and genotyping using next-generation sequencing data. *Nat. Genet.* **43**, 491-498 (2011).
11. Delcher, A. L., Phillippy, A., Carlton, J. & Salzberg, S. L. Fast algorithms for large-scale genome alignment and comparison. *Nucleic Acids Res.* **30**, 2478-2483 (2002).
12. Kurz, S. et al. Versatile and open software for comparing large genomes. *Genome Biol.* **5**, R12 (2004).
13. Croucher, N.J., et al. Rapid phylogenetic analysis of large samples of recombinant bacterial whole genome sequences using Gubbins. *Nucleic Acids Res.* **43**, e15 (2015).
14. Guindon, S. & Gasquel, O. A simple, fast, and accurate algorithm to estimate large phylogenies by maximum likelihood. *Syst. Biol.* **52**, 696-704 (2003).
15. Guindon, S. et al. New algorithms and methods to estimate maximum-likelihood phylogenies: assessing the performance of PhyML 3.0. *Syst. Biol.* **59**, 307-321 (2010).
16. Schwengers, O., et al, A. Bakta: rapid and standardized annotation of bacterial genomes via alignment-free sequence identification. *Microb. Genom.* **7**, 000685 (2021).
17. Page, A. J. et al. Roary: rapid large-scale prokaryote pan genome analysis. *Bioinformatics.* **31**, 3691-3693 (2015).
18. Boutet, E. et al. UniProtKB/Swiss-Prot, the manually annotated section of the UniProt KnowledgeBase: how to use the entry view. *Methods Mol. Biol.* **1374**, 23-54 (2016).
