# Supplementary material for: Genomic insights into the 2020 mass die-off event among African elephants
Source: Nat Commun. 2025 Sep 26;16:8525. doi: 10.1038/s41467-025-63446-7 (PMC12475079; doi:10.1038/s41467-025-63446-7)
Supplement: Supplementary file 2 — Description of Additional Supplementary Files [file 41467_2025_63446_MOESM2_ESM.pdf]

Description of Additional Supplementary Files

File Name: Supplementary Data 1
Description: This file contains digital DNA-DNA hybridization values between the VF20HR genome and type strain genomes in the TYGS database.

File Name: Supplementary Data 2
Description: This file contains detailed information of the 29 Bisgaard taxon 45 and *P. multocida* genomes used in this study.

File Name: Supplementary Data 3
Description: This file contains the tree file for 29 Bisgaard taxon 45 and *P. multocida* genomes in Newick format.

File Name: Supplementary Data 4
Description: This file contains the annotated genome of VH20HR in GenBank Flat File format.

File Name: Supplementary Data 5
Description: This file contains the annotated genome of 20VMG1457/1 in GenBank Flat File format.

File Name: Supplementary Data 6
Description: This file contains the annotated genome of 20VMG1457/5 in GenBank Flat File format.

File Name: Supplementary Data 7
Description: This file contains the annotated genome of VDG05162/2 in GenBank Flat File format.

File Name: Supplementary Data 8
Description: This file contains the annotated genome of VMG0908 in GenBank Flat File format.

File Name: Supplementary Data 9
Description: This file contains the annotated genome of 14589/75 in GenBank Flat File format.

File Name: Supplementary Data 10
Description: This file contains the annotated genome of CDC F 4484 in GenBank Flat File format.

File Name: Supplementary Data 11
Description: This file contains the annotated genome of CDC G 9955 in GenBank Flat File format.

File Name: Supplementary Data 12
Description: This file contains the annotated genome of ATCC BAA-600 in GenBank Flat File format.

File Name: Supplementary Data 13

Description: This file contains the annotated genome of SSI P 876 in GenBank Flat File format.

File Name: Supplementary Data 14
Description: This file contains the annotated genome of HIM 1004-6 in GenBank Flat File format.

File Name: Supplementary Data 15
Description: This file contains information about the 42 genes present in the three elephant VF20/112 isolates but absent in the other Bisgaard taxon 45 isolates.
