# Supplementary material for: Genomic insights into the 2020 mass die-off event among African elephants
Source: Nat Commun. 2025 Sep 26;16:8525. doi: 10.1038/s41467-025-63446-7 (PMC12475079; doi:10.1038/s41467-025-63446-7)
Supplement: Supplementary file 18 — Reporting Summary [file 41467_2025_63446_MOESM18_ESM.pdf]

nature portfolio

Corresponding author(s): Jesper Larsen

Last updated by author(s): Aug 13, 2025

Reporting Summary

Nature Portfolio wishes to improve the reproducibility of the work that we publish. This form provides structure for consistency and transparency in reporting. For further information on Nature Portfolio policies, see our Editorial Policies and the Editorial Policy Checklist.

Statistics

For all statistical analyses, confirm that the following items are present in the figure legend, table legend, main text, or Methods section.

n/a	Confirmed	
[x]	[ ]	The exact sample size (*n*) for each experimental group/condition, given as a discrete number and unit of measurement
[x]	[ ]	A statement on whether measurements were taken from distinct samples or whether the same sample was measured repeatedly
[x]	[ ]	The statistical test(s) used AND whether they are one- or two-sided<br>*Only common tests should be described solely by name; describe more complex techniques in the Methods section.*
[x]	[ ]	A description of all covariates tested
[x]	[ ]	A description of any assumptions or corrections, such as tests of normality and adjustment for multiple comparisons
[x]	[ ]	A full description of the statistical parameters including central tendency (e.g. means) or other basic estimates (e.g. regression coefficient) AND variation (e.g. standard deviation) or associated estimates of uncertainty (e.g. confidence intervals)
[x]	[ ]	For null hypothesis testing, the test statistic (e.g. *F*, *t*, *r*) with confidence intervals, effect sizes, degrees of freedom and *P* value noted<br>*Give P values as exact values whenever suitable.*
[x]	[ ]	For Bayesian analysis, information on the choice of priors and Markov chain Monte Carlo settings
[x]	[ ]	For hierarchical and complex designs, identification of the appropriate level for tests and full reporting of outcomes
[x]	[ ]	Estimates of effect sizes (e.g. Cohen's *d*, Pearson's *r*), indicating how they were calculated

Our web collection on statistics for biologists contains articles on many of the points above.

Software and code

Policy information about availability of computer code

Data collection	No software was used to collect data in this study.
Data analysis	TYGS server<br>Dorado v.7.2.1<br>Hybracter v.0.7.3<br>SPAdes v.3.15<br>NASP v.1.2.0<br>Gubbins v.3.3.1<br>PhyML v.3.0<br>Bakta v.1.8.2<br>Roary v.3.13.0

For manuscripts utilizing custom algorithms or software that are central to the research but not yet described in published literature, software must be made available to editors and reviewers. We strongly encourage code deposition in a community repository (e.g. GitHub). See the Nature Portfolio guidelines for submitting code & software for further information.

Data

Policy information about availability of data

All manuscripts must include a data availability statement. This statement should provide the following information, where applicable:

- Accession codes, unique identifiers, or web links for publicly available datasets
- A description of any restrictions on data availability
- For clinical datasets or third party data, please ensure that the statement adheres to our policy

dDDH values between the VF20HR genome and type strain genomes in the TYGS database are listed in Supplementary Data 1. Detailed information of the 29 Bisgaard taxon 45 and P. multocida genomes used in this study is listed in Supplementary Data 2. Sequence data generated in this study have been deposited in the European Nucleotide Archive/NCBI Sequence Read Archive under BioProject PRJNA1204292, and the accession numbers are provided in Supplementary Data 2. Sequence data from other sources comprised the VF20HR genome (GenBank accession no. JAQAHH000000000.1) and the genomes of the five P. multocida isolates expressing capsular serogroup E (GenBank accession no. NZ_JAMJVB000000000.1, NZ_JAMJVC000000000.1, NZ_JAMJVD000000000.1, NZ_JAMJVE000000000.1, and NZ_JAMJVF000000000.1). The phylogeny shown in Fig. 1 is provided as a Newick file in Supplementary Data 3. The annotated Bisgaard taxon 45 genomes are provided in GenBank Flat File format in Supplementary Data 4-14. The 42 genes present in the three elephant VF20/112 isolates but absent in the other Bisgaard taxon 45 isolates are listed in Supplementary Data 15.

Research involving human participants, their data, or biological material

Policy information about studies with human participants or human data. See also policy information about sex, gender (identity/presentation), and sexual orientation and race, ethnicity and racism.

Reporting on sex and gender	This study did not involve human participants.
Reporting on race, ethnicity, or other socially relevant groupings	This study did not involve human participants.
Population characteristics	This study did not involve human participants.
Recruitment	This study did not involve human participants.
Ethics oversight	This study did not involve human participants.

Note that full information on the approval of the study protocol must also be provided in the manuscript.

Field-specific reporting

Please select the one below that is the best fit for your research. If you are not sure, read the appropriate sections before making your selection.

☒ Life sciences ☐ Behavioural & social sciences ☐ Ecological, evolutionary & environmental sciences

For a reference copy of the document with all sections, see nature.com/documents/nr-reporting-summary-flat.pdf

Life sciences study design

All studies must disclose on these points even when the disclosure is negative.

Sample size	We did not calculate the sample size as our study was observational rather than experimental. The sample size was determined by the availability of isolates collected in other studies and by veterinaríans in South Africa.
Data exclusions	No data were excluded from our analysis.
Replication	We used 100 bootstrap replicates to reconstruct the phylogeny.
Randomization	We did not perform randomization as our study was observational rather than experimental.
Blinding	We did not perform bliinding as our study was observational rather than experimental.

Reporting for specific materials, systems and methods

We require information from authors about some types of materials, experimental systems and methods used in many studies. Here, indicate whether each material, system or method listed is relevant to your study. If you are not sure if a list item applies to your research, read the appropriate section before selecting a response.

Materials & experimental systems

n/a	Involved in the study
☒	☐ Antibodies
☒	☐ Eukaryotic cell lines
☒	☐ Palaeontology and archaeology
☐	☒ Animals and other organisms
☒	☐ Clinical data
☒	☐ Dual use research of concern
☒	☐ Plants

Methods

n/a	Involved in the study
☒	☐ ChIP-seq
☒	☐ Flow cytometry
☒	☐ MRI-based neuroimaging

Animals and other research organisms

Policy information about studies involving animals; ARRIVE guidelines recommended for reporting animal research, and Sex and Gender in Research

Laboratory animals	The study did not involve laboratory animals.
Wild animals	The study involved elephant VF20/112 isolates from Zimbabwe as well as cheetah, lion, and cattle isolates from South Africa.
Reporting on sex	Determination of sex was not relevant in this study.
Field-collected samples	This study did not involve active collection of field samples. The elephant VF20/112 isolates from Zimbabwe as well as the cheetah, lion, and cattle isolates from South Africa were detected in diagnostic samples that were submitted from veterinarians to Vetdiagnostix, Blue Hills, Midrand, South Africa. The animals were not specifically selected for this study and were under veterinary treatment for other reasons.
Ethics oversight	Samples collected for diagnostic purposes do not require ethical approval in South Africa.

Note that full information on the approval of the study protocol must also be provided in the manuscript.

Plants

Seed stocks	*Report on the source of all seed stocks or other plant material used. If applicable, state the seed stock centre and catalogue number. If plant specimens were collected from the field, describe the collection location, date and sampling procedures.*
Novel plant genotypes	*Describe the methods by which all novel plant genotypes were produced. This includes those generated by transgenic approaches, gene editing, chemical/radiation-based mutagenesis and hybridization. For transgenic lines, describe the transformation method, the number of independent lines analyzed and the generation upon which experiments were performed. For gene-edited lines, describe the editor used, the endogenous sequence targeted for editing, the targeting guide RNA sequence (if applicable) and how the editor was applied.*
Authentication	*Describe any authentication procedures for each seed stock used or novel genotype generated. Describe any experiments used to assess the effect of a mutation and, where applicable, how potential secondary effects (e.g. second site T-DNA insertions, mosiacism, off-target gene editing) were examined.*
